# Melatonin Alleviates Venous Dysfunction in a Mouse Model of Iliac Vein Occlusion

**DOI:** 10.3389/fimmu.2022.870981

**Published:** 2022-05-02

**Authors:** Zhiye Guo, Xiaolong Du, Yu Zhou, Dandan Xu, Xingyu Xu, Shan Lu, Feng Ran

**Affiliations:** ^1^ Key Laboratory of Cardiovascular and Cerebrovascular Medicine, Collaborative Innovation Center for Cardiovascular Disease Translational Medicine, Nanjing Medical University, Nanjing, China; ^2^ Department of Vascular Surgery, Nanjing Drum Tower Hospital, The Affiliated Hospital of Nanjing University Medical School, Nanjing, China

**Keywords:** iliac vein occlusion, melatonin, venous permeability, venous inflammatory response, venous dysfunction

## Abstract

The iliac vein can be severely stenosed and occluded due to thrombosis, tumor compression, or an anatomical abnormality. Such occlusion could result in limb swelling, venous claudication, and persistent leg ulcers. Its devastating sequelae heavily impact patients lifestyles and the social economy. Due to a lack of a stable and easy-to-operate iliac vein occlusion (IVO) model, its underlying molecular mechanism and pathophysiological process has not been completely understood. Melatonin (MLT) plays a critical role in anti-inflammation, but the potential protective effect of melatonin on venous dysfunction induced by IVO has not been revealed. In this study, a mouse model of IVO was established to study the effects of MLT on injured veins. The results of laser speckle images and Evans blue showed that MLT inhibited venous permeability in an IVO mouse model. Furthermore, MLT suppressed inflammation of surrounding tissues close to the affected vein by inhibiting the mRNA levels of TNF-α, IL-1α, and MCP-1. In addition, endothelial injury was inhibited by MLT using zonula occludens protein-1 (ZO-1) staining. Taken together, we elucidated the therapeutic effect of MLT on vascular dysfunction induced by IVO, mainly by inhibiting the TNF-α, IL-1α, and MCP-1 mRNA levels, improving endothelial function, and inhibiting vascular leakage.

## Introduction

Iliac vein occlusion (IVO) could be the result of thrombosis or external compression of the vein and is more common on the left side (1). Iliac vein compression syndrome (IVCS), also known as May-Thurner syndrome or Cockett syndrome, is mainly caused by extrinsic compression (2, 3). It shows common traits of disturbance of lower extremities and pelvis venous reflux. In severe IVCS, acute deep vein thrombosis (DVT) of lower extremities and various forms of chronic venous diseases (CVD) will be observed (4). It has been reported that more than 70% of the compression of the left common iliac vein is related to left DVT (5). Chronic venous obstruction may lead to long-term diseases, including intractable swelling and pain. Patients with this disease suffered from chronic pain and even lifestyle changes, which could cause unemployment and greatly affect society (1, 6).

Long-term venous occlusion induced by venous hypertension can cause a series of pathological changes, including impairing valve and endothelial functions, inducing an inflammatory response, and even resulting in post-thrombotic syndrome (7, 8). When the shear stress of blood flow changes, it induces inflammation response, endothelial cell activation, and leukocyte adhesion, activation, and infiltration. With disease progression, endothelial permeability increases gradually, contributing to the vascular contents infiltrating the surrounding tissues (9). Eventually, venous deterioration, dysfunction, and inflammation are aggravated. Furthermore, it is believed that pro-inflammatory factors such as TNF-α, IL-1α, and MCP-1 can be released during inflammation (10), so these factors were selected as indicators in our study. Although there have been some theories about the correlation between IVO and venous hypertension, little is known about the underlying molecular mechanism.

Melatonin (MLT), a ubiquitous molecule, can be synthesized by the pineal glands in vertebrates as an endocrine hormone (11). According to previous reports, MLT shows various effects, including anti-inflammation, antioxidation, anti-tumor, and circadian rhythm regulation. It has been proved to be beneficial for cerebrovascular diseases and cognitive decline associated with aging and diabetes (11–13). In terms of anti-inflammation, MLT can reduce pro-inflammatory cytokines, adipokines, chemokines, and adhesion molecules (14). Additionally, it serves as a direct free radical scavenger to neutralize different free radicals (15) and also reduces oxidative stress *via* mitochondrial function improvement. IVO is known to be associated with vascular and tissue inflammation. Thus, we explored the molecular mechanism of melatonin underlying the treatment of vascular dysfunction in the IVO model. In previous studies, the common doses of MLT were 10 μg/ml (16), 15 μg/ml (17, 18), and 25 μg/ml (19). Thus, the dose of 10 μg/ml was taken in this study.

Here, we demonstrated that MLT reduced the affected muscular inflammation around vessels in the IVO model using quantitative real-time PCR (qRT-PCR). The results of Laser speckle imaging, Evans blue staining, and ZO-1 immunofluorescence staining validated the idea that MLT inhibits vascular leakage and maintains endothelial stability. Taken together, this study demonstrates the protective effects of MLT on venous leakage, providing a new idea for the treatment of venous dysfunction in the future.

## Materials and Methods

### Mice

C57BL/6 background male mice at the age of 8 weeks were obtained from the Animal Core Facility of Nanjing Medical University in this study. All animal procedures were approved by the Animal Care Committee of Nanjing Medical University (approval numbers NJMU-2102034) in accordance with institutional animal care and use guidelines.

### Mouse Model of Iliac Vein Occlusion

IVO was induced by the iliac vein ligation as described previously by das Gracas et al. (20) on hamsters. Mice were anesthetized with 4% isoflurane mix gas for 30 s in a supine position, and anesthesia was continuously given with 1%–1.5% isoflurane mix gas during the surgery. The hair of the left lower abdominal quadrant and the leg was shaved cleanly to expose the surgical area entirely. A groin incision was made to carefully expose the left external iliac vein. The left external iliac vein was separated from the artery and ligated with a 7-0 polypropylene suture. The skin was then closed by a 4-0 polypropylene suture. Furthermore, the same operation was performed without vein ligation in sham groups. All mice were divided into four groups as follows: sham/normal saline (NS; *n* = 5), sham/melatonin (MLT; *n* = 5), IVO/NS (*n* = 5), and IVO/MLT (*n* = 10). For the drinking water, MLT (#73-31-4, MCE) was dissolved in 0.1% ethanol and the final MLT concentration was maintained at 10 μg/ml (16). Vehicle groups were given drinking water with 0.1% ethanol.

### Laser Speckle Image-Based Tissue Perfusion Measurement

**Figure 1:**
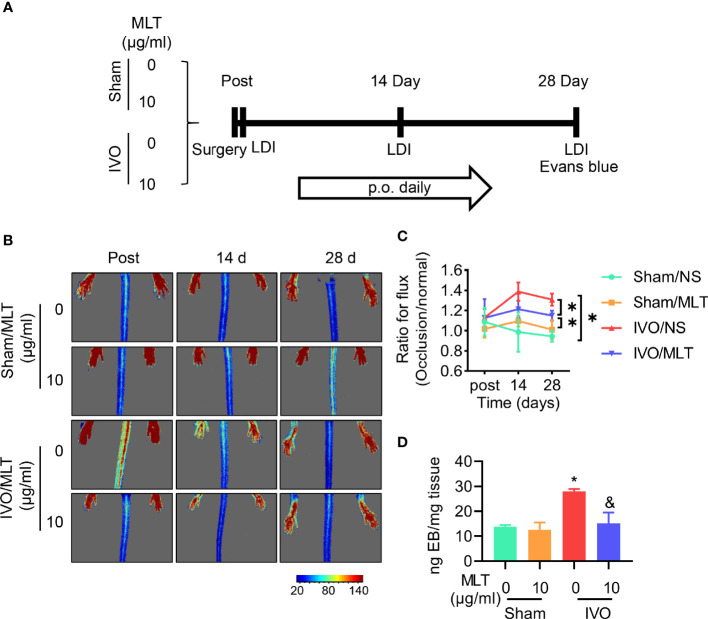
MLT inhibits permeability in a mouse model of iliac vein occlusion (IVO). **(A)** Schematic representation of experimental procedure. p.o., per os. **(B, C)** The blood flow perfusion of ligated or normal hindlimb was detected after surgery (post), 14 days (14 d), and 28 days (28 d) after surgery using the laser speckle system. Sham/normal saline (NS; *n* = 5), sham/MLT (*n* = 5), IVO/NS (*n* = 5), and IVO/MLT (*n* = 10). **(B)** Representative perfusion images were shown. **(C)** Quantitative analysis of blood perfusion with the ratio of left (occlusion) to right (normal). *p < 0.05 **(D)** Quantitative content of Evans blue in semimembranosus (SM) muscle of mice. Sham/NS (*n* = 4), sham/MLT (*n* = 4), IVO/NS (*n* = 4), and IVO/MLT (*n* = 5). *p < 0.05 vs. sham/NS, ^&^ *p* < 0.05 vs. IVO/NS. Data presented as mean ± SD.

The blood flow perfusion of ligated or normal hindlimb was obtained by using a laser speckle imaging system (moor FLPI-2; Moor Instruments) as described previously (21). Mice were fixed on a 37°C heating pad in a supine position. The hindlimb blood flow was measured immediately after surgery (post), and 14 days and 28 days after surgery (**Figure 1A**). The region of interest was ensured to cover both the hindlimb paws. The final perfusion was expressed by the ratio of left (occlusion) to right (normal) limb (22).

### Evans Blue

Mice were injected with 0.5% Evans blue (#E2129, Sigma-Aldrich, Shanghai, China) through the tail vein and allowed to circulate for 1 h. The mouse’s skin and nose will turn blue within 10 s if the injection is successful (23). The mice were then sacrificed by cervical dislocation and given intracardiac perfusion with normal saline. The semimembranosus (SM) muscle tissue was removed. The muscle was weighed separately and 500 μl of formamide (#F810079, Macklin, Shanghai, China) was added to incubate for 24–48 h in a 55°C water bath to extract Evans Blue from tissue (24). After the formamide/Evans blue mixture was centrifuged, the OD values of the supernatant were measured at 620 nm. The content of Evans blue per milligram of tissue based on the standard curve of Evans blue in formamide was calculated.

### Immunofluorescence Staining

The gastrocnemius (GC) muscles were excised from mice and immersed in TISSUE TEK O.C.T compound (#4583, Sakura, USA) for cryo-embedding. Five-micrometer cross-sections were cut on slides. The slicing was fixed in 4% paraformaldehyde for 15 min at room temperature and rinsed three times in PBS for 5 min each to remove OCT. Then, the slicing was blocked with 10% bovine serum albumin (BSA) (Sigma, Burlington, MA, United States) for 1 h. Aspirated BSA solution and the sections were incubated with Anti-ZO1 tight junction (#61-7300, Invitrogen) overnight. CoraLite488-conjugated Affinipure Goat Anti-Rabbit IgG(H+L) (#SA00013-2, Proteintech) was used as fluorochrome-conjugated secondary antibody, incubated for 1 h at 37°C from light, and nuclear stained with DAPI (#P0131, Beyotime). The representative magnified images were captured with Confocal microscopy (CarlZeiss LSM710; Carl Zeiss) at 20× magnification under the same brightness/contrast setting.

### RNA Extraction and Quantitative Real-Time PCR

For the gastrocnemius muscle, the total RNA was extracted using a Trizol reagent (R401-01, Vazyme, Nanjing, China), and reverse transcribed into complementary DNA (cDNA) using an RT SuperMix (R323-01, Vazyme, China) following the manufacturer’s protocol. Quantitative Real-Time PCR (qRT-PCR) assays were conducted with a SYBR Green Mix (Q131-02, Vazyme, China) on the QuantStudio 5 (Applied Biosystems, Thermo Fisher Scientific). The related gene-specific primers (TNF-α, IL-1α, and MCP-1) used are listed in **Supplementary Table 1**. The number of target genes was normalized to β-actin using the 2^−ΔΔCt^ method. The mRNA expression of TNF-α, IL-1α, and MCP-1 was normalized to that of the sham/NS group.

### Statistics

All data were presented as the mean ± SD. Statistical analysis was performed with GraphPad Prism 8 using a one-way analysis of variance (ANOVA) or Student’s *t*-test. The differences among groups were analyzed by a one-way ANOVA using Tukey’s multiple comparison post-hoc test. *p* < 0.05 was considered statistically significant.

## Results

### Melatonin Inhibits Permeability in Mouse Model of IVO

After successfully establishing the IVO model, the therapeutic effects of MLT were evaluated. The results of the laser speckle imaging system showed that MLT has no effect on hindlimb blood perfusion in sham groups. Moreover, MLT did not affect the blood perfusion ratio between the ligated side and the healthy side at the early stage but increased the blood perfusion ratio of the hindlimb at 14 and 28 days after the operation. After MLT treatment, the blood perfusion ratio of bilateral hindlimbs decreased (**Figures 1B, C**).

In **Figure 1D**, the content of Evans blue in muscular tissue increased significantly after iliac vein ligation. Consistent with previous laser speckle imaging, this staining result suggests that there is higher leakage of the dye from the vasculature in IVO mice. Under physiological conditions, the endothelium is impermeable to albumin and is often used to detect vascular permeability *in vivo*. Thus, our results indicate that iliac vein ligation led to blood stasis in the hindlimb, venous pressure elevation, and loss of tight contact with partial endothelial cells. After MLT administration, the leakage of Evans blue in tissue was decreased and vascular leakage was effectively alleviated. The above results show the increased endothelial permeability in the IVO mouse model to be consistent with the development of IVCS.

### Melatonin Relieves Affected Muscular Inflammatory Response in IVO Mice

**Figure 2:**
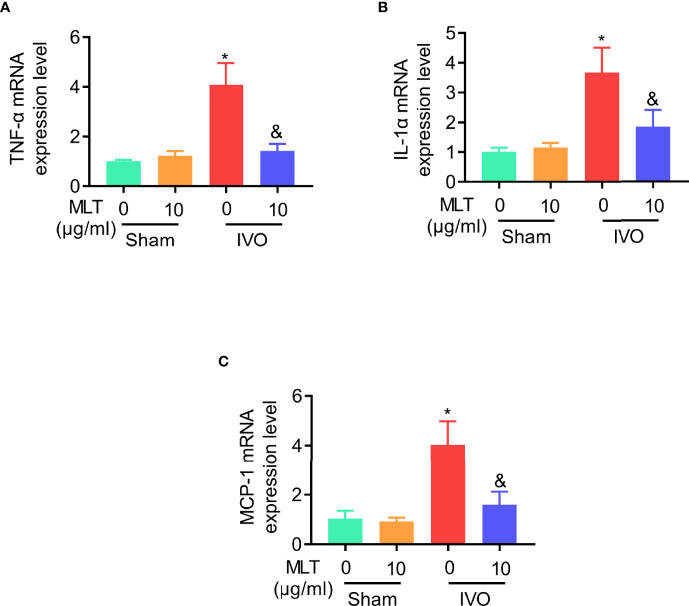
MLT inhibits affected muscular inflammatory response in an IVO model. **(A–C)** Relative mRNA expression of TNF-α **(A)**, IL-1α **(B)**, and MCP-1 **(C)** in gastrocnemius muscle was measured using real-time PCR. β-actin mRNA was used as an internal control, and expression was normalized to that of animals in sham/NS group. Sham/NS (*n* = 4), sham/MLT (*n* = 4), IVO/NS (*n* = 4), and IVO/MLT (*n* = 6). Values are presented as means ± SD. *p < 0.05 vs. sham/NS, ^&^ *p* < 0.05 vs. IVO/NS.

Since IVO relates to inflammation in clinic, the levels of inflammatory cytokines, including TNF-α, IL-1α, and MCP-1, were detected in the gastrocnemius muscle from IVO mice. The results of qRT-PCR showed that the level of inflammatory cytokines in the IVO group was higher than in sham groups. However, these highly expressed inflammatory factors were effectively suppressed after the administration of melatonin (**Figures 2A–C**). In general, the intervention of MLT plays an anti-inflammatory role in the IVO model and has a protective effect on affected muscles.

### Melatonin Prevents Endothelial Dysfunction

**Figure 3:**
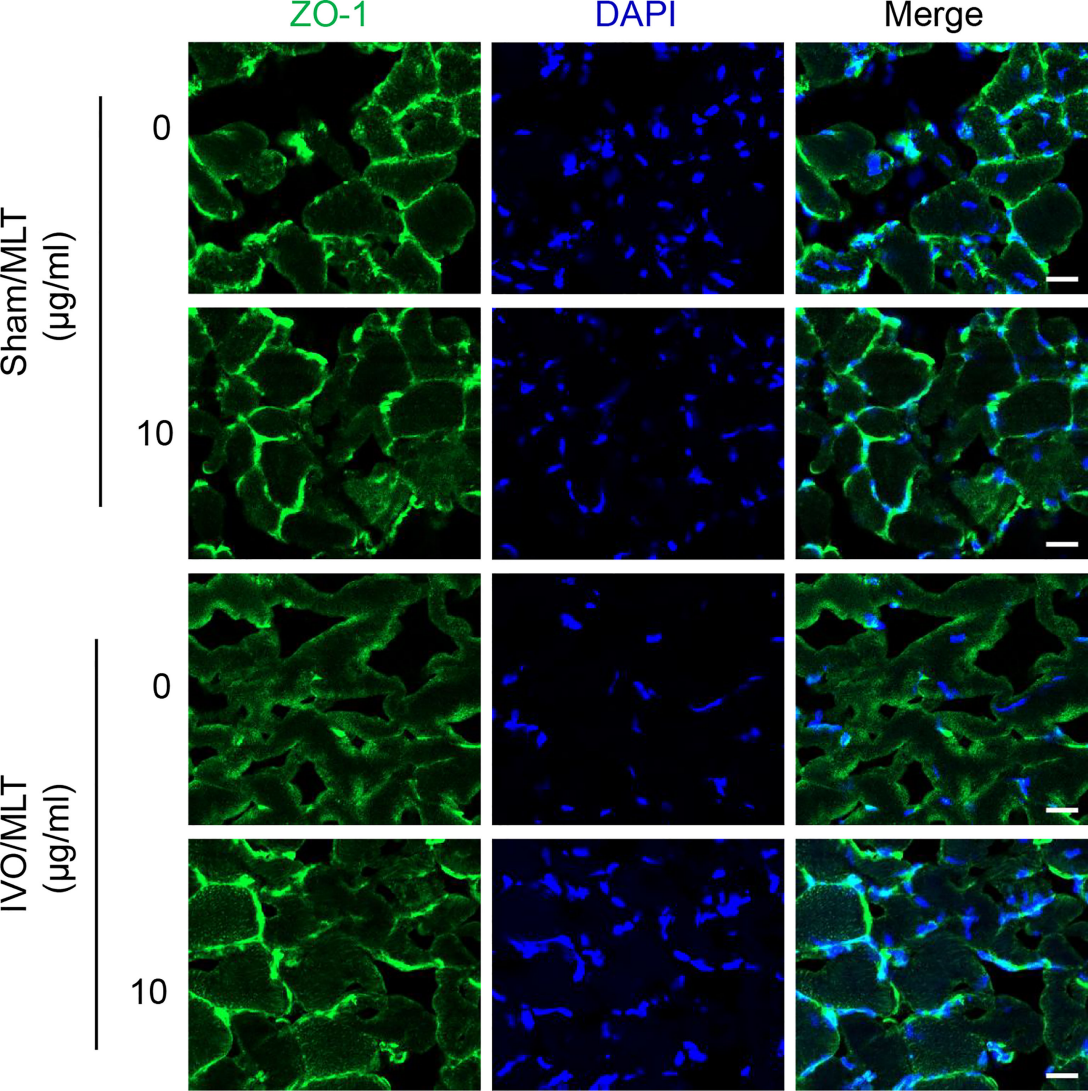
MLT prevents endothelial dysfunction in IVO model. Representative immunofluorescence staining images with ZO-1 (green) using gastrocnemius muscle from mice (scale bar: 20 μm).

Gastrocnemius muscle near the damaged vein was harvested and used for ZO-1 immunofluorescence staining to test the effect of MLT on vascular leakage (**Figure 3**). Under physiological conditions, the connection between gastrocnemius muscle fascicles was not affected. In contrast, 28 days after iliac vein ligation, the fluorescence intensity at the junction between muscle fascicles was weak and muscle fascicles were incomplete. Melatonin showed a protective effect on vascular leakage. After 28 days of continuous treatment, the fluorescence intensity at the junction was enhanced, suggesting that the connections between muscle fascicles were repaired. Also, the continuous fluorescent signal along the cell edges indicated muscle fascicles were complete after MLT treatment. Briefly, these results suggest that in the IVO mouse model, the connections between muscular cells are damaged because of vascular leakage and MLT could maintain intercellular connections implying reduced vascular leakage and improved endothelial junctions.

## Discussion

Based on the protective therapeutic role of MLT on cardiovascular diseases (19), we explored the effect of MLT on venous endothelial cells and closed skeletal muscle in IVO. Our results showed that MLT relieved tissue inflammation adjacent to the ligated vein by inhibiting the expression of TNF-α, IL-1α, and MCP-1. Moreover, it reduced the foot perfusion ratio in mice, maintained the tight junction of vascular endothelium and perivascular muscular tissue, and reduced venous leakage.

MLT maintains venous endothelial function in the IVO model. Generally, MLT has a profound protective role for endothelial function in arteries or capillaries. A previous study by Hu et al. showed that MLT ameliorates vascular endothelial dysfunction by suppressing the TLR4/NF-κB system in high-fat-fed rabbits (25). The same group also confirmed that MLT inhibits macrophage infiltration and promoted plaque stabilization by upregulating the anti-inflammatory HGF/c-Met system in the atherosclerotic rabbit (26). In a cardiac ischemia/reperfusion-related study by Hao Zhou et al., they showed that MLT improves microvascular function *via* activation of platelet PPARγ (27). Zhang et al. (28), using *Caspase1/11-/-* mice, *Il1r-/-* mice, and melatonin-treated mice, revealed that MLT reduces hypoxia-induced pulmonary vascular endothelial leakage in the pulmonary arterial hypertension (PAH) model, related to the effects of inflammasome-associated endothelial permeability. Moreover, it also showed that MLT reduces the activation of endothelial cells. Herein, we focused on exploring the therapeutic role of MLT in venous endothelial function.

Furthermore, MLT suppresses the injury of surrounding skeletal muscle caused by venous leakage. IVO causes venous hypertension, resulting from venous obstruction-induced blockage of venous return. It is believed that venous hypertension caused by vein ligature could lead to venous valve damage over time and reflux, which further aggravate venous hypertension and tissue damage (20). Moreover, venous hypertension causes venous leakage, accompanied by the release of pro-inflammatory factors and cells, including cytokines, neutrophils, and macrophages. As reported by Takase et al., the number of granulocytes, monocytes, and macrophages is increased around the affected venous wall, while the expression of intercellular adhesion molecules such as ICAM-1 is increased (29). The released factors and cells lead to the response in closed skeletal muscles, damaging their physiological function. Compared to the sham/NS group, the increase of lower limb blood perfusion and the elevated expression of inflammatory factors (TNF-α, IL-1α, and MCP-1) in the muscle around the affected vein were observed in the IVO model. Also, the tight junction of the muscle was destroyed after iliac vein ligation. In contrast, after the treatment of MLT, the above situation has been improved. It indicated that MLT suppresses the injury of adjacent skeletal muscle caused by venous leakage in the IVO model.

The main limitation of this study is the inadequate number of mice for each group. More mice are needed to confirm the protective role of MLT on IVO in future studies. Also, the mouse model of IVO using ligation is not sufficient to represent IVO in clinic. Venous acute stasis was induced by iliac vein ligation, which was slightly different from the vascular occlusion caused by chronic iliac vein compression. A long-term vein stenosis model could be constructed without endothelial injury to mimic the progress of IVCS. Thus, a further modified mouse model for IVO is needed.

In this study, we identified that MLT alleviates venous endothelial leakage in an IVO mouse model. Mechanistically, in the IVO model, MLT suppresses the inflammatory response to maintain venous endothelial function. This indicated that MLT could be considered a potential treatment for IVO.

## Data Availability Statement

The original contributions presented in the study are included in the article/**Supplementary Material**. Further inquiries can be directed to the corresponding authors.

## Ethics Statement

The animal study was reviewed and approved by the Animal Care and Use Committee of Nanjing Medical University.

## Author Contributions

FR and SL designed the study. ZG and XD conducted the searches. YZ, DX, and XX analyzed the data. FR, XD, and ZG wrote the manuscript. All authors contributed to the article and approved the submitted version.

## Conflict of Interest

The authors declare that the research was conducted in the absence of any commercial or financial relationships that could be construed as a potential conflict of interest.

## Publisher’s Note

All claims expressed in this article are solely those of the authors and do not necessarily represent those of their affiliated organizations, or those of the publisher, the editors and the reviewers. Any product that may be evaluated in this article, or claim that may be made by its manufacturer, is not guaranteed or endorsed by the publisher.
