# Supplementary figures and images for: Melatonin Alleviates Venous Dysfunction in a Mouse Model of Iliac Vein Occlusion
Source: Front Immunol. 2022 May 2;13:870981. doi: 10.3389/fimmu.2022.870981 (PMC9108156; doi:10.3389/fimmu.2022.870981)

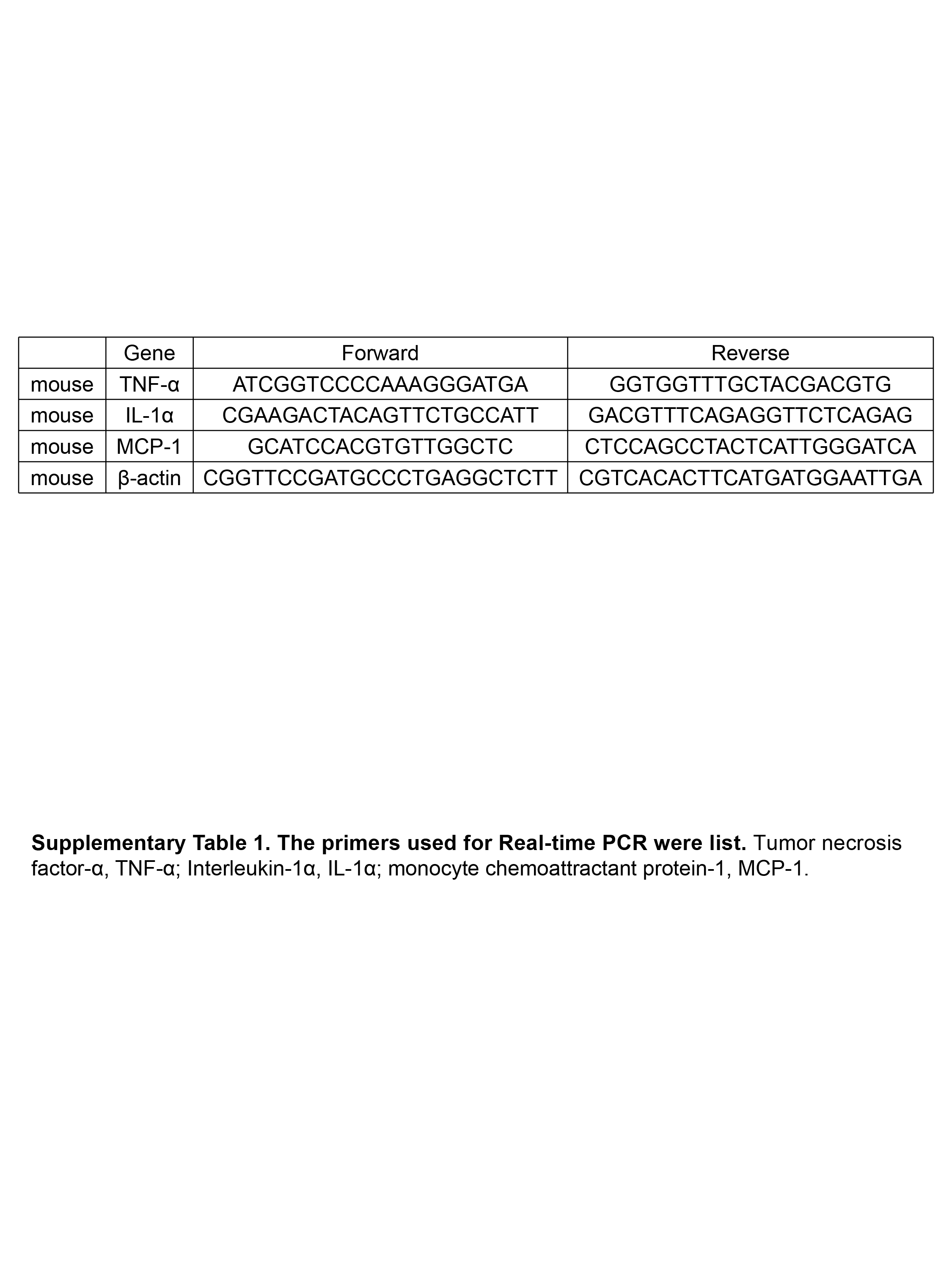

Supplement: Supplementary file 1 [file Image_1.tif]
